# Experimental Study on Mechanical Properties and Durability of Polymer Silica Fume Concrete with Vinyl Ester Resin

**DOI:** 10.3390/ma16020757

**Published:** 2023-01-12

**Authors:** Hosein Zanjirani Farahani, Atiye Farahani, Pouyan Fakharian, Danial Jahed Armaghani

**Affiliations:** 1 Department of Civil Engineering, Tafresh University, Tafresh P.O. Box 39518-79611, Iran; 2 Department of Construction Engineering and Management, Energy Institute of Higher Education, Saveh P.O. Box 39177-67746, Iran; 3 Department of Urban Planning, Engineering Networks and Systems, Institute of Architecture and Construction, South Ural State University, 454080 Chelyabinsk, Russia

**Keywords:** polymer silica fume concrete, vinyl ester resin, tensile strength, compressive strength, durability, water absorption

## Abstract

Polymer concrete, which contains silica fume powder and vinyl ester resin as two replacements for Portland cement, has improved mechanical properties and durability compared to ordinary concrete. Thus, this kind of concrete is considered to be a high-strength concrete that is resistant to corrosion and chemical attacks. In this paper, the effects of the combination of silica fume powder and vinyl ester resin as two Portland cement replacements on the workability and slump value, initial and final water absorption, compressive and tensile strength, and failure and fracture paths of the polymer concrete have been investigated. All investigations have been based on 16 different polymer concrete mixture designs. The results indicate that the optimum percentages for a combination of silica fume and vinyl ester resin, which has the maximum compressive strength (34.26 MPa) and the maximum tensile strength (4.92 MPa), are a combination of 10% silica fume and 5% vinyl ester resin. To evaluate the durability of polymer concrete, the water absorption of all mixture designs has also been measured. Accordingly, the mixture design, which includes a combination of 15% vinyl ester resin and 5% silica fume, has a minimum initial and final water absorption equal to 0.62% and 1.95%, respectively.

## 1. Introduction

There is a significant amount of waste plastic in our natural environment, and this is a concern on a global scale [1,2]. Concrete is the material that is utilized the most in building and has a reputation for being easy to deteriorate. The life duration of concrete is shortened when it is subjected to external attacks such as sulfate attacks, alkali–silica reactions, corrosion, and drying shrinkage. As a consequence of these assaults, the service life of the target is shortened and the requirement for early repair and maintenance is increased; this, in turn, leads to increased life cycle costs and structural failures [3,4].

Throughout their service lives, many structures made of reinforced concrete may be subject to material deterioration, unanticipated overloading that can cause physical damage, cyclic temperature changes, and chemical attack as a result of the harsh climatic conditions that they are exposed to. The mixture design proportions, placement, compaction, and curing procedures [5,6] all play a role in determining the durability, strength, stiffness, and workability of concrete, as well as a number of other attributes [7,8,9,10]. In recent years, the use of resins in polymer concrete mixture design has increased to improve the properties of the fresh concrete and hardened concrete. With the use of polymer concrete, it is possible to apply different materials and techniques to prepare environmentally friendly concrete [11,12,13,14,15].

In this context, a novel approach is the utilization of resin as an alternative to Portland cement in the production of concrete [16,17]. The application of glass fiber reinforced plastics (GFRP) has seen a substantial uptick in popularity during the past few years. Because of its great strength and resilience to corrosion, glass fiber reinforced plastic (GFRP) is a good choice for use in a wide variety of applications [16].

In the context of the construction industry, the term “polymer concrete” refers to the use of a polymer, such as epoxy, polyester, or vinyl ester materials, as a coating, supplement, or cement replacement in order to improve the mechanical and durability properties of concrete. In general, the rate at which strength is developed in polymer concrete is significantly faster than that of conventional concrete. It is utilized in the production of high-strength concrete, which is a well-known substance that is resistant to corrosion and chemicals [17]. In addition, fillers and frequently other cementitious materials are added in order to adjust the ensuing strength development and mechanical properties. This is completed in order to get the desired effect. Polymer concrete has the potential to be a substantial alternative to OPC-based concrete or to operate as a supplement in precast structures, strengthening, and repair [4,17,18]. This is because polymer concrete is impermeable, in addition to having other potentials.

On the basis of the content that was examined, a composition of vinyl ester polymer concrete as well as the results of experimental tests that were conducted to evaluate the fundamental mechanical properties of the material is presented. Following the strategy for sustainable development in the construction industry, the material cost of polymer concrete was reduced by lowering the consumption of raw materials and partially replacing the micro filler fraction with a recycled waste product known as calcium fly ash [17]. This brought the total cost of the material down to a lower level. When combined with other fillers and cementitious materials, resins can produce a cost-effective composite material that improves the physicomechanical and durability properties of concrete structures [4,19]. This is especially true when resins are used in combination with other fillers and cementitious materials. The research demonstrated that the concrete-damaged plasticity material model may be utilized well for the purpose of describing the behavior of PC mechanical components [19,20].

An investigation on the viability of repurposing broken glass from old lighting fixtures as an aggregate for polymer concrete was conducted and detailed in an article. The findings of the research have demonstrated that the aggregate that is obtained from waste glass can, in fact, be utilized fruitfully in the manufacturing of polymer concrete [21,22]. The application of super absorbent polymers (SAP), also referred to simply as SAP, is a technique that has proven to be extraordinarily effective in reducing the amount of autogenous shrinkage (AS) that takes place in high-performance concrete [23,24]. This technique is also sometimes referred to as SAP. Analyses and research were conducted on the impacts of the SAP content and the inclusion method on the flowability, mechanical characteristics, shrinkage performance, and microstructure of internally cured concrete. When added to the SAP, preabsorbed water has the potential to delay early cement hydration, enhance the pace of later cement hydration as well as the final hydration degree, and improve the strength of the concrete. The concrete’s shrinkage can be efficiently improved by combining a SAP with preabsorbed water, and this combination produces a shrinkage reduction impact that is more pronounced than the effect produced by simply adding the SAP dry. Concrete can be efficiently prevented from cracking if a SAP is added to it [23,24,25,26]. This will enhance the microstructure of the concrete, which will improve its density as well as its resistance to cracking.

Conventionally, polymer concrete is a type of concrete in which resin plays a role as a replacement for Portland cement in the cement paste [27,28]. For this purpose, different resins such as polyester resin, vinyl ester, epoxy, etc., are used in polymer concrete [29].

Epoxy resin is one of the applications of resins in polymer concrete. Epoxy resin can be used as a replacement for Portland cement or fine aggregates. Due to better environmental performance, the use of polymer concretes is mostly for non-structural purposes in industry, such as the lining of man-holes, septic tanks, etc. [30,31,32,33].

Today, the use of polymers in ordinary cement concrete has become more important and attractive in order to reduce the consumption of Portland cement in the field of civil engineering and to preserve the environment [32]. Additionally, research results have indicated that the use of epoxy in concrete will improve the durability of concrete and increase the corrosion resistance of concrete in the hot marine environment [33]. In addition, polymer concrete containing vinyl ester is used as an overlay on the decks of concrete bridges. The vinyl ester overlay is more expensive and more difficult to transport than polyester overlays [34]. However, vinyl ester has a lower cost than epoxy resin [35]. In this regard, Peters [36] noted that in contrast to other thermosets, vinyl ester does not have to sacrifice its resilience to heat and chemicals in order to attain better flexibility and toughness. In addition, in comparison to polyester, vinyl ester has a lower ester content and a lower saturation, so it shrinks less during the sintering process, has a lower calorific value during the firing process, and is more resistant to hydrolysis. These advantages can be attributed to the fact that vinyl ester is a lower-density material.

In addition, Lokuge and Aravinthan [37] have conducted research on the compressive and tensile strengths of polymer mortar. In their study, they employed mixture designs that contained two different types of vinyl ester resin and epoxy resin coupled with fly ash and sand. The results showed that the compressive strengths of polymer mortar containing epoxy and polymer mortar containing vinyl ester were 75 and 113 MPa, respectively. Additionally, the ultimate strains of polymer mortar containing epoxy and polymer mortar containing vinyl ester were 8% and 4%, respectively. The indicated tensile strength for both types of polymer mortar was 15 MPa. The results showed that the optimal amount of polymer varies from 12 to 13%.

The durability of concrete structures can be improved by including silica fume in the cement mixture. This will also extend their usable life. Therefore, the incorporation of silica fume into the cementitious components of concrete helps to lessen the capillary porosity and preserves the level of cement hydration that has already been achieved. As a consequence of this, the microstructure of the interfacial transition zone (ITZ) is improved by silica fume concrete, which in turn leads to a reduction in diffusion [38,39]. To achieve the same results as Portland cement in terms of the improvement of the mechanical characteristics of concrete [40,41,42], silica fume and nano-silica are also utilized as pozzolans.

In this paper, the effect of silica fume powder and vinyl ester resin as two Portland cement replacements in polymer concrete has been investigated. All investigations were based on 16 polymer concrete mixture designs. In the experimental studies of this paper, workability, initial and final water absorption, durability, compressive, and tensile strengths of polymer concrete mixture designs have been investigated. Additionally, the optimum percentages for a combination of silica fume and vinyl ester resin in a concrete mixture design are presented.

**Figure 1:**
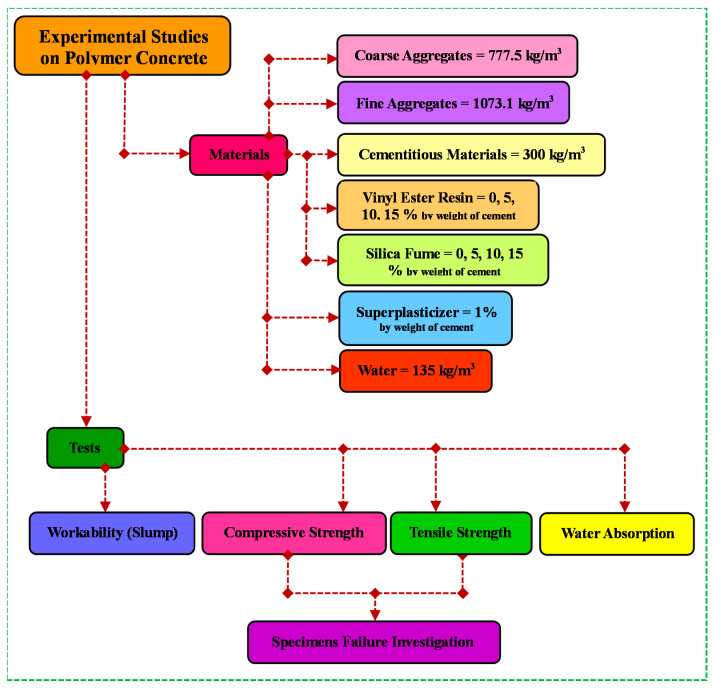
Framework of this paper.

An overview of the framework for this study is presented in Figure 1.

## 2. Experimental Studies

In this section, the materials used in the preparation of the mixture design, the mixture proportions, and the process of preparation, casting, and curing of the specimens have been elaborated.

### 2.1. Materials

The Portland cement used in this study is type II Portland cement, which was supplied by Neyzar Qom Cement Company, Qom Province, Iran. The specific gravity and blain of type II Portland cement are 3.18 g/cm^3^ and 3200 cm^2^/g, respectively. The chemical composition of Portland cement is summarized in Table 1.

The silica fume (SF) used as a replacement for Portland cement was supplied by the Iran Ferrosilicon Company, Semnan, Iran. The silica fume is a non-crystalline, amorphous type that can participate in the chemical reaction in concrete and combine with the lime in the cement paste to form an adhesive gel and to some extent reduce the alkalinity of the concrete environment. Its particle size is less than 45 microns. The specific gravity and blain of the silica fume are 2.14 g/cm^3^ and 4200 cm^2^/g, respectively. The chemical composition of the silica fume is summarized in Table 2.

In this study, to reduce cement consumption, which reduces the release of CO_2_ gas from the cement production process and helps the environment, the silica fume has been used as a replacement for Portland cement in the concrete mixture design. Thus, in the mixture design, by including the silica fume at its optimal percentage, the slump and workability of the fresh concrete decreased and the compressive and tensile strengths increased.

To crosslink the vinyl ester resin in the mixture design, Methyl Ethyl Ketone Peroxide (MEKP) as a hardener in the amount of 1.5% by weight of the vinyl ester resin, Cobalt Naphthenate as an accelerator in the amount of 0.4% by weight of the vinyl ester resin, and Dimethyl Aniline (DMA) as an auxiliary accelerator in the amount of 0.4% by weight of the vinyl ester resin should be added to it at average temperatures. The properties of the vinyl ester resin are summarized in Table 3.

The crushed limestone aggregates used in this research were provided from the Khorabad Village, Kahak Section, Qom Province, Iran, and graded according to ASTM C33. The maximum size of the coarse aggregates is 12.5 mm. The specific gravity and absorption value are 2.71 and 2.73% for the coarse aggregates, and 2.65 and 1.01% for the fine aggregates, respectively.

Additionally, to improve the workability of the fresh concrete, Polycarboxylate Ether Polymer superplasticizer was employed, which was supplied by the Shimi Sakhteman Company, Tehran, Iran. Water used for the mixing and processing of concrete specimens in this research was drinking water from the city of Qom, Iran.

### 2.2. Mixture Proportions

In this study, various proportions of the mixtures were produced by changing the vinyl ester resin contents (5%, 10%, and 15%) and the silica fume contents (5%, 10%, and 15%). The mixture proportions for 1 m^3^ concrete and slump values of these 16 concrete mixture designs are summarized in Table 4.

### 2.3. Preparation, Casting, and Curing of Specimens

**Figure 2:**
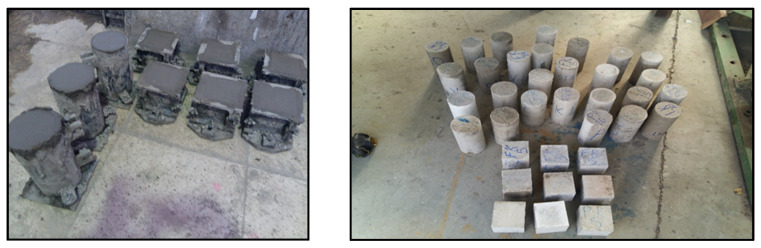
Preparation and casting of specimens.

In the laboratory in the Markazi Province of Iran, the concrete mixtures were made for the purpose of undergoing tests to determine their compressive strength, tensile strength, and water absorption. Cast in steel molds, the concrete specimens for the compressive strength and water absorption tests were cubes measuring 100 mm on a side and 100 mm on a height, while the specimens for the tensile strength test were cylinders measuring 100 mm on a diameter and 200 mm on a height. Both types of specimens were compacted on a vibrating table. After being cast for twenty-four hours, the specimens were demolded and cured in water for twenty-eight days at a temperature of twenty-one degrees Celsius and a relative humidity of thirty percent (Figure 2).

### 2.4. Test Methods

#### 2.4.1. Water Absorption Test

**Figure 3:**
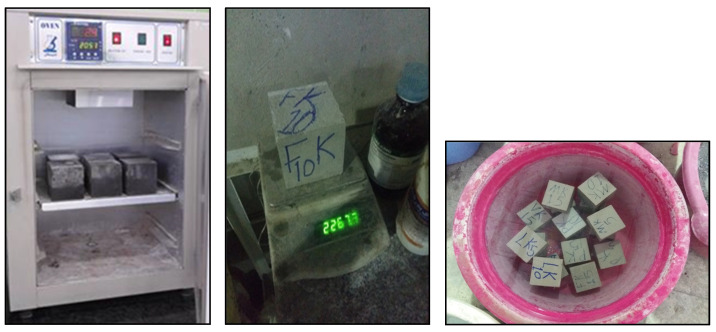
Polymer concrete specimens in oven, in water, and weighing for water absorption test.

The initial and final water absorption tests of concrete were performed according to ASTM C642. After 28 days, the specimens were placed in an oven at a temperature of 110–100 °C to reach a stabilized weight and dry thoroughly. They were then weighed, their dry weight recorded, and then placed in a tub of water. In order to test the initial water absorption, the specimens were taken out of the tub after 30 min, and after drying their surfaces with a cloth, they were weighed and the results recorded. To test the final water absorption, the specimens were returned to the tub for 48 h and then removed from the water. Subsequently, the surface of the samples was dried, and their weight was again measured and recorded (Figure 3). Finally, to calculate the amount of water absorption, the following equation was used:

(1) $$\mathrm{Water}\mathrm{Absorption}\left(\%\right)=\frac{M-M_{0}}{M_{0}}\times 100$$

where *M*_0_ is the weight of the dried concrete inside the oven and *M* is the weight of the concrete after placing it in the water.

#### 2.4.2. Compressive Strength Test

**Figure 4:**
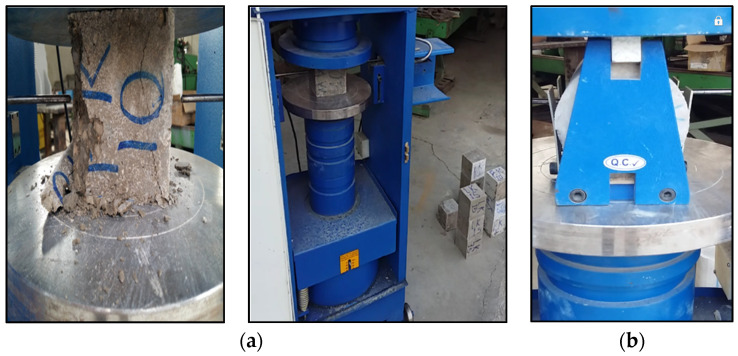
(**a**) Compressive strength test apparatus; (**b**) Tensile strength test apparatus.

The compressive strength of concrete is a significant parameter in the design of reinforced concrete structures that determines the properties of concrete. In this study, the compressive strength test was performed according to the ASTM C39 standard (Figure 4a). This standard applies to concrete with a specific gravity of more than 800 kg/m^3^. Concrete cylindrical specimens measuring 150,300 mm were recommended in accordance with this standard. The application of axial load continued until the moment of failure of the specimen and the appearance of the rupture surface, in addition to reducing the compressive load. The results of this experiment depend on the specimen size, design, method of mixing, specimen age, temperature, sampling method, and humidity at the time of processing. According to this standard, the speed and rate of loading are 0.2 to 0.3 MPa per second, continuously. The compressive strength obtained in this study is based on the cubic specimen strength of 100 mm × 100 mm × 100 mm. Hence, this value has been converted to the 150 mm × 150 mm × 150 mm standard cubic strength and then to the equivalent strength of the standard cylinder specimen with 150 mm diameter and 300 mm height. These experiments have been performed on the specimens for 28 days.

#### 2.4.3. Tensile Strength Test

In this study, the tensile strength of the specimens has been obtained according to the Brazilian test method and the ASTM C496M (Figure 4b). Due to the high tensile strength of steel reinforcement, the tensile strength of concrete is neglected in most concrete structure designs. However, it should be noted that the tensile strength of concrete has a significant role in reducing cracking and increasing the durability of reinforced concrete structures.

The maximum load that can be tolerated by a concrete specimen depends on the geometric dimensions of the specimen, which determine the tensile strength of concrete. Therefore, the most effective and important criteria in determining the durability of concrete are its water absorption and tensile strength.

In the polymer concrete prepared in this study, due to the use of the vinyl ester resin and silica fume as replacements for Portland cement, segregation in the aggregates as well as bleeding have occurred. Accordingly, the fine aggregates have gone to the top of the mold, and the coarse aggregates have gone to the bottom of the mold. Compaction, vibration, and concrete surface polishing became difficult and needed more time for molding. It is worth mentioning that with the increase in the silica fume in the mixture design, molding and surface polishing of the specimens became difficult due to the decrease in hydration temperature. With the increased use of the vinyl ester resin, due to the adhesion and crosslinking of vinyl ester during mixing, molding and surface polishing of specimens became difficult.

## 3. Result and Discussion

In this section, the results of the tests to determine the percentage of water absorption, compressive strength, and tensile strength of polymer concrete specimens containing different percentages (by weight of cement) of the silica fume and vinyl ester resin have been analyzed.

### 3.1. Water Absorption Results

**Figure 5:**
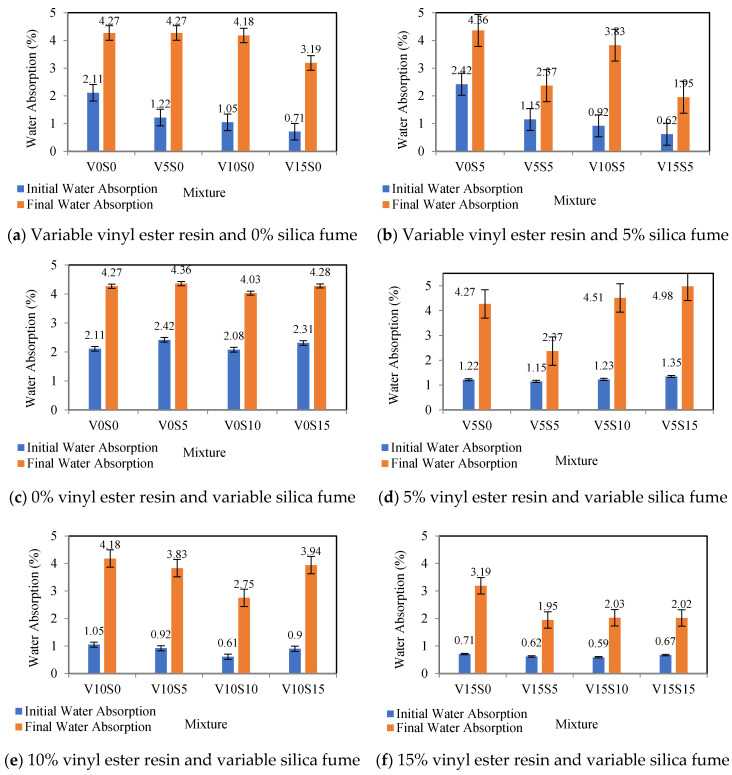
Water absorption of polymer concrete mixtures.

In this experiment, the amount of water absorption for 30 min is the same as the initial water absorption, and the 48 h absorption is the same as the final water absorption. Table 5 and Figure 5 present the water absorption percentages of control polymer concrete specimens, specimens with the vinyl ester resin, specimens with the silica fume, and specimens with the combination of the vinyl ester resin and silica fume at different percentages. After curing, different percentages of the vinyl ester resin and silica fume as two replacements for Portland cement have a significant effect on the initial and final water absorption of concrete.

As indicated in Table 5 and Figure 5a, in the mixture design containing the vinyl ester resin and without the silica fume, by increasing the vinyl ester resin from 0 to 15%, the initial and final water absorption percentages decreased by 66.35% and 25.29%, respectively. As seen in Table 5 and Figure 5c, in the mixture design containing the silica fume and without the vinyl ester resin, by increasing the silica fume from 0 to 10%, the initial and final water absorption percentages decreased by 1.42% and 5.62%, respectively. The results of Table 5, Figure 5b,f indicate that the mixture design, including a combination of 15% vinyl ester resin and 5% silica fume, has a minimum initial and final water absorption equal to 0.62% and 1.95%, respectively. The results of Table 5 and Figure 5d indicate that the mixture design, including a combination of 5% vinyl ester resin and 5% silica fume, has a minimum initial and final water absorption equal to 1.15% and 2.37%, respectively. The results of Table 5 and Figure 5e indicate that the mixture design, including a combination of 10% vinyl ester resin and 10% silica fume, has a minimum initial and final water absorption equal to 0.61% and 2.75%, respectively.

Finally, the maximum final water absorption (4.98%) is related to the mixture design containing a combination of 5% vinyl ester resin and 15% silica fume, and the minimum final water absorption (1.95%) is related to the mixture design containing a combination of 15% vinyl ester resin and 5% silica fume. Furthermore, the maximum initial water absorption (2.42%) is related to the mixture design containing a combination of 0% vinyl ester resin and 5% silica fume, and the minimum initial water absorption (0.61%) is related to the mixture design containing a combination of 10% vinyl ester resin and 10% silica fume. The vinyl ester resin causes adhesion and stiffness in the mixture design, and silica fume reduces the slump and workability of the mixture design. Accordingly, they should be combined with a suitable percentage of superplasticizer to ensure the workability of the concrete. The addition of the silica fume to the mixture causes a combination of silica particles with calcium hydroxide crystals and produces calcium silicate gel, which over time fills the pores in the cement paste and microstructure in the concrete and compacts the concrete structure. It also fills the pore spaces between the aggregates and the cement paste.

The amount of initial water absorption is greatest when the percentage of resin in the mixture is at its lowest. This is because the vinyl ester resin has the property of increasing adhesion and stiffness in the mixture design. The results show that this occurs when the percentage of resin in the mixture is at its lowest. In addition, the highest possible level of water absorption can be accomplished by using a resin content of 5% of the total. According to the findings, the application of the silica fume does not have a substantial impact, either initially or ultimately, on the amount of water that is absorbed. According to the findings, there is not a discernible difference in the amount of water absorption that takes place when the percentage of the vinyl ester resin and silica fume is set at 5%. The quantity of initial water absorption drops by 47% when the amount of the vinyl ester resin and silica fume is reduced to 10%; nevertheless, the quantity of final water absorption rises by 16% when this is performed. Additionally, the initial and final water absorption both decrease by 42% and 15%, respectively, if there is a shift of 15% in the percentage of the vinyl ester resin and silica fume. This represents a 10% increase in the amount of resin and silica fume compared to the first scenario.

### 3.2. Compressive and Tensile Strength Results

As indicated in Table 4 and Table 5, in the specimen containing 15% silica fume and without vinyl ester resin, the slump value of the fresh concrete is 78 mm, which is 5 mm less than the control concrete slump. The compressive strength of this specimen compared to the control concrete has been reduced by 6.15 MPa. Its tensile strength has also decreased by 0.91 MPa compared to the control concrete.

Furthermore, in the specimen containing 10% silica fume and without the vinyl ester resin, the slump value of the fresh concrete is 80 mm, which is 3 mm less than the control concrete slump. The compressive strength of this specimen compared to the control concrete has increased by 4.04 MPa. Its tensile strength has also increased by 1.71 MPa compared to the control concrete. Therefore, the optimum percentage of silica fume as a replacement for Portland cement is 10%.

In the specimen containing 15% vinyl ester resin and without the silica fume, the slump value of the fresh concrete is 78 mm, which is 5 mm less than the control concrete slump. The compressive strength of this specimen compared to the control concrete has been reduced by 11.75 MPa. Its tensile strength has also decreased by 2.98 MPa compared to the control concrete.

Therefore, the slump value of the fresh concrete in the specimen that contains 10% vinyl ester resin but does not contain the silica fume is 82 mm, which is one millimeter less than the slump of the control concrete. This specimen has a compressive strength that is 3.99 MPa lower than the control concrete when compared to the concrete used as the standard. In comparison to the concrete that served as the control, its tensile strength has likewise dropped by 1.31 MPa. The slump value of new concrete measured 85 mm in the specimen that included 5% vinyl ester resin but did not contain the silica fume; this value was 2 mm higher than the slump measured in the control concrete. When compared to the control concrete, this specimen has a compressive strength that is 6.12 MPa lower than the control concrete. In comparison to the concrete that served as the control, its tensile strength has likewise decreased by 0.64 MPa.

The slump value of the fresh concrete in the specimen that contains a mixture of 10% silica fume and 5% vinyl ester resin is 80 mm, which is 3 mm smaller than the slump of the control concrete, which contains 0% silica fume and 0% vinyl ester resin. As a direct consequence of this, the control specimen’s compressive strength has been raised by 0.61 MPa when compared to the specimen under investigation. In comparison to the concrete that served as the control, its tensile strength has also increased by 0.07 MPa. Because of this, the formulation of this mixture is the best possible combination of the silica fume and vinyl ester resin that can be found in the polymer concrete.

Accordingly, in the specimen containing a combination of 15% silica fume and 15% vinyl ester resin, the slump value of the fresh concrete was 82 mm, which is 1 mm less than the control concrete slump. Therefore, the compressive strength of this specimen has decreased by 14.39 MPa compared to the control concrete. Its tensile strength has also decreased by 3.49 MPa compared to the control concrete.

**Figure 6:**
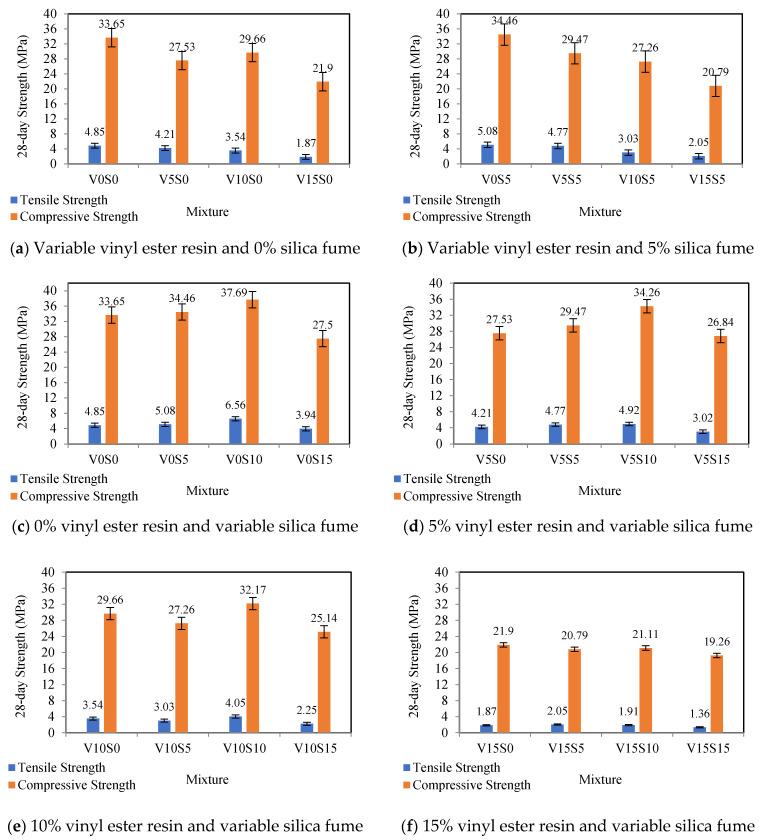
Compressive and tensile strength of polymer concrete mixtures.

Figure 5 indicates the column charts of the influence of the silica fume and vinyl ester resin as the replacements for Portland cement on the 28-day compressive and tensile strengths of polymer concretes. Among the results of the strength of all of the specimens, according to Table 5 and Figure 6a–f, the specimen containing 10% silica fume and without the vinyl ester resin has the maximum compressive strength (37.69 MPa) and the maximum tensile strength (6.56 MPa). In addition, among the results of the strength of all of the specimens, according to Table 5 and Figure 6f, the specimen containing a combination of 15% silica fume and 15% vinyl ester resin has the minimum compressive strength (19.26 MPa) and the minimum tensile strength (1.36 MPa).

According to Table 5 and Figure 6c, among the specimens containing only silica fume, the specimen containing 15% silica fume has the minimum compressive strength (27.50 MPa) and the minimum tensile strength (3.94 MPa). According to Table 5 and Figure 6a, among the specimens containing only the vinyl ester resin, the specimen containing 10% vinyl ester resin has the maximum compressive strength (29.66 MPa), and the specimen containing 5% vinyl ester resin has the maximum tensile strength (4.21 MPa). Additionally, according to Table 5 and Figure 6a, among the specimens containing only the vinyl ester resin, 15% vinyl ester resin has the minimum compressive strength (21.90 MPa) and the minimum tensile strength (1.87 MPa).

Therefore, the optimum percentages of the vinyl ester resin, which has the maximum compressive strength (29.66 MPa) and the maximum tensile strength (4.21 MPa), are 10% and 5%, respectively. The optimum 10% silica fume has the maximum compressive strength (37.69 MPa) and the maximum tensile strength (6.56 MPa). Hence, the optimum percentages for a combination of the silica fume and vinyl ester resin, which provide the maximum compressive strength (34.26 MPa) and the maximum tensile strength (4.92 MPa), are a combination of 10% silica fume and 5% vinyl ester resin.

### 3.3. Failure of Specimens in the Compressive and Tensile Strength Tests

**Figure 7:**
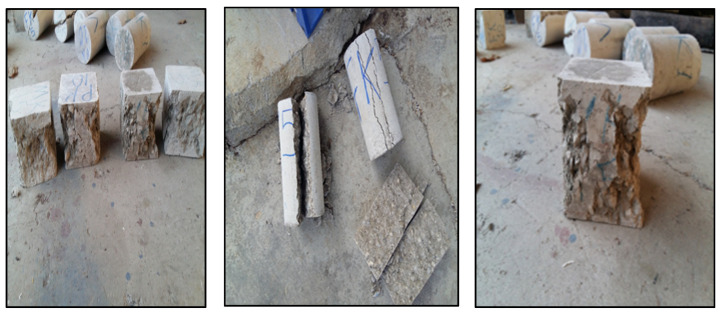
Failure of polymer concrete specimens during compressive and tensile strength tests.

Figure 7 presents the failure of the polymer concrete specimens during the compressive and tensile strength tests. As indicated in Figure 7, during the loading of the concrete specimens in the compressive strength test, shear cracks have diagonally appeared. Unlike the ordinary concrete, in the concrete specimens containing the vinyl ester resin and silica fume, more fracture paths pass through the aggregates because, in polymer concrete, the strength of the cement paste and the boundary area between the aggregates and cement paste are greater than the strength of the aggregates. As a result, due to its lower vinyl ester resin strength compared to its silica fume strength, the vinyl ester resin is more effective in controlling and limiting the strength of concrete.

## 4. Conclusions

Based on laboratory observations and the results of various experiments performed on the polymer concrete specimens in this study, the results are summarized as follows:

(I) In the mixture design containing the vinyl ester resin and without the silica fume, by increasing the vinyl ester resin from 0 to 15%, the initial and final water absorption percentages decrease by 66.35% and 25.29%, respectively.
(II) In the mixture design containing the silica fume and without the vinyl ester resin, by increasing the silica fume from 0 to 10%, the initial and final water absorption percentages decrease by 1.42% and 5.62%, respectively.
(III) Among the results of the initial and final water absorption of all of the specimens, the mixture design including a combination of 15% vinyl ester resin and 5% silica fume has the minimum initial and final water absorption equal to 0.62% and 1.95%, respectively.
(IV) Among the results of the strengths of all of the specimens, the specimen containing 10% silica fume has the maximum compressive strength (37.69 MPa) and the maximum tensile strength (6.56 MPa).
(V) The optimum percentages of the vinyl ester resin, which have the maximum compressive strength (29.66 MPa) and the maximum tensile strength (4.21 MPa), are 10% and 5%, respectively.
(VI) The optimum percentage of the silica fume, which has the maximum compressive strength (37.69 MPa) and the maximum tensile strength (6.56 MPa), is 10%.
(VII) The optimum percentages for the combination of the silica fume and the vinyl ester resin, which have the maximum compressive strength (34.26 MPa) and the maximum tensile strength (4.92 MPa), are a combination of 10% silica fume and 5% vinyl ester resin.
(VIII) In the specimen containing a combination of 10% silica fume and 5% vinyl ester resin, the slump value of the fresh concrete is 80 mm, which is 3 mm less than the slump value of the control concrete (0% silica fume and 0% vinyl ester resin).
(IX) During the loading of the concrete specimens in the compressive strength test, shear cracks have appeared diagonally. Unlike the ordinary concrete, in the concrete specimens containing the vinyl ester resin and the silica fume, more fracture paths pass through the aggregates.

## Figures and Tables

**Table 1:** Chemical composition of type II Portland cement.

Property	Value
SiO_2_ (%)	20.70
Al_2_O_3_ (%)	5.20
Fe_2_O_3_ (%)	4.60
CaO (%)	63.85
MgO (%)	1.80
SO_3_ (%)	2.20
K_2_O (%)	0.50
Na_2_O (%)	0.15
LOI (%)	1.00

**Table 2:** Chemical composition of silica fume.

Property	Value
SiO_2_ (%)	89.22
Al_2_O_3_ (%)	1.20
Fe_2_O_3_ (%)	2.12
CaO (%)	1.64
MgO (%)	1.61
K_2_O (%)	1.054
Na_2_O (%)	0.556
LOI (%)	2.60

**Table 3:** Properties of vinyl ester resin.

Property	Value
Type Liquid state	Bisphenol
Viscosity (MPas @250c)	420–580
Gel time (min)	35–45
Density (g/cm^3^)	1.29

**Table 4:** Concrete mixture composition (per 1 m^3^ concrete).

Mixture	Vinyl Ester (%) ^(I)^	Silica Fume (%) ^(I)^	Water (kg/m^3^)	Cement (kg/m^3^)	W/CM ^(II)^	Fine Aggregates (kg/m^3^)	Coarse Aggregates (kg/m^3^)	Superplasticizer (%) ^(I)^	Slump (mm)
V0S0	0	0	135	300	0.45	1073.1	777.5	1	83
V5S0	5	0	135	285	0.45	1073.1	777.5	1	85
V10S0	10	0	135	270	0.45	1073.1	777.5	1	82
V15S0	15	0	135	255	0.45	1073.1	777.5	1	78
V0S5	0	5	135	285	0.45	1073.1	777.5	1	82
V0S10	0	10	135	270	0.45	1073.1	777.5	1	80
V0S15	0	15	135	255	0.45	1073.1	777.5	1	78
V5S5	5	5	135	270	0.45	1073.1	777.5	1	83
V5S10	5	10	135	255	0.45	1073.1	777.5	1	80
V5S15	5	15	135	240	0.45	1073.1	777.5	1	77
V10S5	10	5	135	255	0.45	1073.1	777.5	1	81
V10S10	10	10	135	240	0.45	1073.1	777.5	1	80.5
V10S15	10	15	135	225	0.45	1073.1	777.5	1	78
V15S5	15	5	135	240	0.45	1073.1	777.5	1	78
V15S10	15	10	135	225	0.45	1073.1	777.5	1	81
V15S15	15	15	135	210	0.45	1073.1	777.5	1	82

^(I)^ % by weight of cement; ^(II)^ Cementitious Materials (including cement, silica fume, and vinyl ester resin).

**Table 5:** Water absorption, tensile, and compressive strength results of specimens.

Mixture	Specimen Weight after Leaving Oven	Specimen Weight after Leaving Water					Percentage of Water Absorption of Concrete Specimens at Different Times (%)					Tensile Strength-28 Days (MPa)	Compressive Strength-28 Days (MPa)
		30 min	60 min	6 h	24 h	48 h	30 min	60 min	6 h	24 h	48 h		
V0S0	2221.7	2268.6	2280.4	2304.8	2315.1	2316.6	2.11	2.64	3.74	4.20	4.27	4.85	33.65
V5S0	2227.2	2254.4	2262.7	2289.5	2316.0	2322.2	1.22	1.59	2.80	3.99	4.27	4.21	27.53
V10S0	2137.0	2159.5	2166.9	2190.6	2217.4	2226.4	1.05	1.40	2.51	3.76	4.18	3.54	29.66
V15S0	2088.5	2103.4	2107.5	2120.6	2145.2	2155.2	0.71	0.91	1.54	2.71	3.19	1.87	21.90
V0S5	2205.6	2258.9	2271.8	2291.2	2300.0	2301.7	2.42	3.00	3.88	4.28	4.36	5.08	34.46
V0S10	2269.5	2316.6	2328.7	2349.5	2358.7	2360.9	2.08	2.61	3.53	3.93	4.03	6.56	37.69
V0S15	2205.3	2256.2	2268.7	2287.7	2296.9	2299.7	2.31	2.87	3.74	4.15	4.28	3.94	27.50
V5S5	2205.7	2231.0	2236.0	2245.0	2253.7	2257.9	1.15	1.37	1.78	2.18	2.37	4.77	29.47
V5S10	2207.1	2234.2	2242.7	2268.4	2297.9	2306.7	1.23	1.61	2.78	4.11	4.51	4.92	34.26
V5S15	2077.7	2105.8	2114.3	2140.4	2172.0	2181.1	1.35	1.76	3.02	4.54	4.98	3.02	26.84
V10S5	2145.9	2165.7	2171.3	2189.1	2216.9	2228.0	0.92	1.18	2.01	3.31	3.83	3.03	27.26
V10S10	2096.3	2109.1	2114.0	2125.9	2143.3	2153.9	0.61	0.84	1.41	2.24	2.75	4.05	32.17
V10S15	2049.7	2068.2	2073.2	2089.7	2118.5	2130.5	0.90	1.15	1.95	3.36	3.94	2.25	25.14
V15S5	2175.5	2188.9	2193.3	2204.6	2213.4	2217.9	0.62	0.82	1.34	1.74	1.95	2.05	20.79
V15S10	2121.7	2134.2	2138.8	2151.5	2161.5	2164.7	0.59	0.81	1.40	1.88	2.03	1.91	21.11
V15S15	2109.7	2123.9	2127.9	2138.9	2149.0	2152.4	0.67	0.86	1.38	1.86	2.02	1.36	19.26

## Data Availability

All data, models, or codes that support the findings of this study are available from the corresponding author upon reasonable request.
